# Supplementary material for: Engaging and disengaging recurrent inhibition coincides with sensing and unsensing of a sensory stimulus
Source: Nat Commun. 2017 May 23;8:15413. doi: 10.1038/ncomms15413 (PMC5457525; doi:10.1038/ncomms15413)
Supplement: Supplementary Information — Supplementary Figures and Supplementary Reference [file ncomms15413-s1.docx]

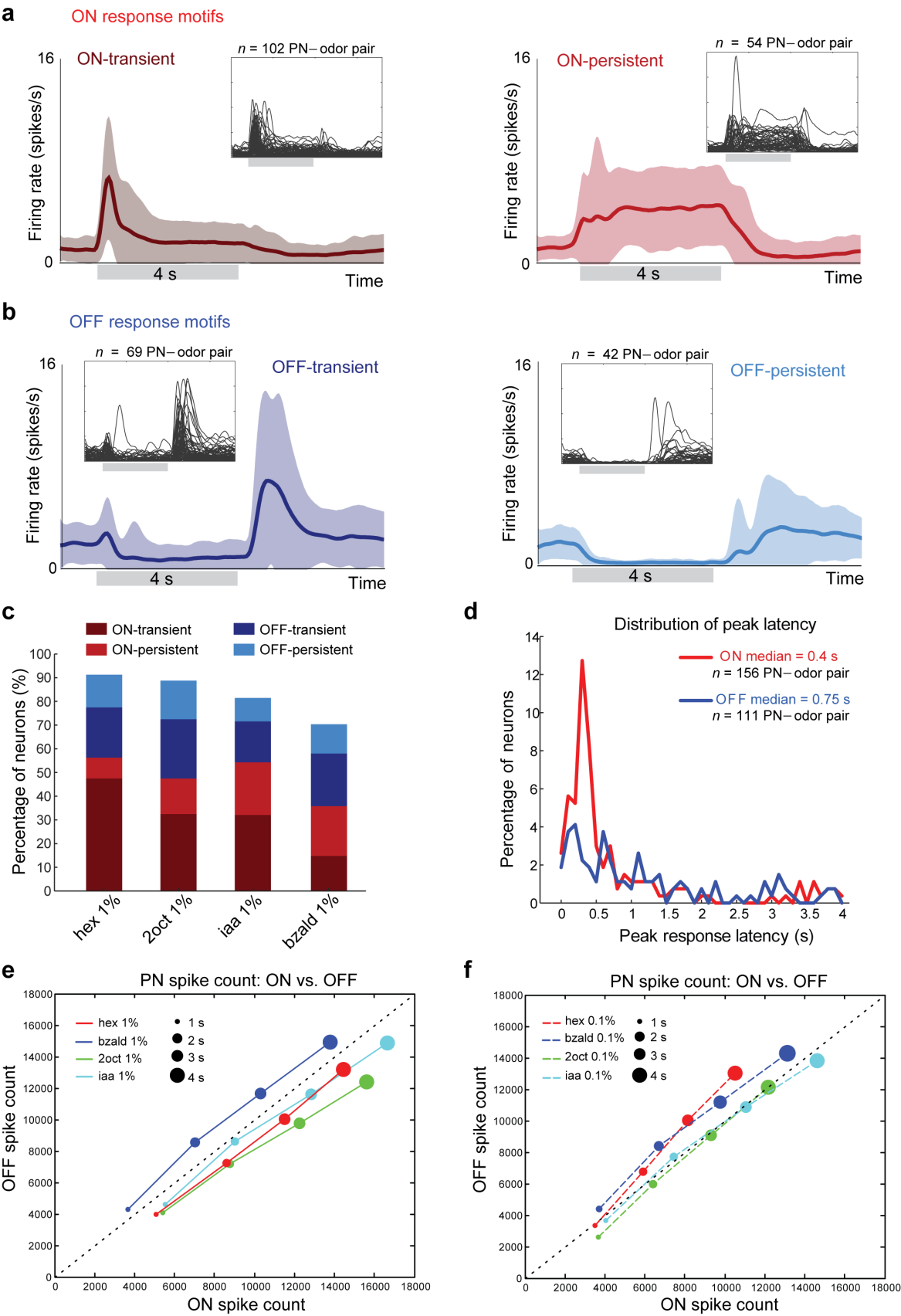


**Supplementary Figure 1: ON vs. OFF response features. (a,b)** Results from an unsupervised clustering analysis of projection neuron responses are summarized and shown here (see Methods for details). Two predominant response types (ON vs. OFF responses) were identified with two major sub-types for each case. Mean firing rates (± s.d.) averaged across projection neurons are shown as a function of time for each response cluster. Insets show all individual PN responses assigned to a cluster. *n* indicates number of PN responses assigned to a cluster. **(c)** Percentages of neurons with a particular response motif: ON transient, ON persistent, OFF transient, OFF persistent are shown for four odorants: hex 1%, 2oct 1%, iaa 1%, bzald 1%. **(d)** The time-to-peak-response distributions for ON and OFF PNs are shown here (see Methods). **(e)** Total PN spike count (summed over all neurons recorded, *n* = 80 for hex and 2oct; *n* = 81 for bzald and iaa) during stimulus ON and stimulus OFF periods are shown for all four odorants used in the study (at 1% v/v). Comparison between ON and OFF spike counts are provided for the following integration window sizes: 1s, 2s, 3s, 4s (beginning from stimulus onset or stimulus offset). Parity between the ON and OFF responses is shown as a dotted line along the diagonal. **(f)** Similar plot as in **panel e** but shown for the lower concentrations (0.1% v/v) of the same four odorants.


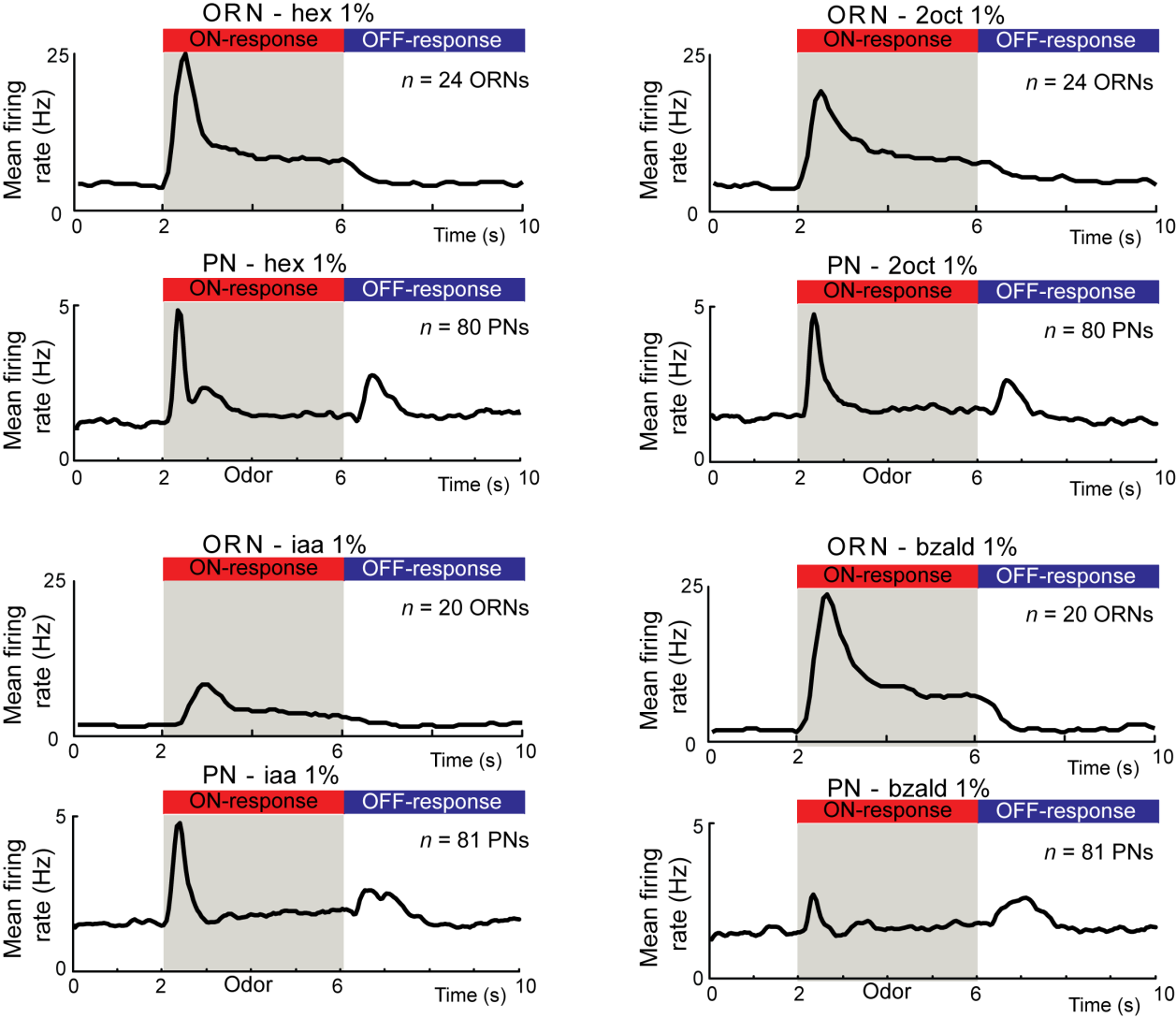


**Supplementary Figure 2: OFF responses are stronger in the antennal lobe.** Odor-evoked mean ensemble firing rates for both ORNs and PNs to the four odors used in the study are shown. ORN mean firing rates are reproduced from^1^. *n* represents total number of neurons recorded for each odor. Gray box indicates 4 s long odor stimulation window. ON and OFF periods are identified with red and blue bars at the top of each plot.


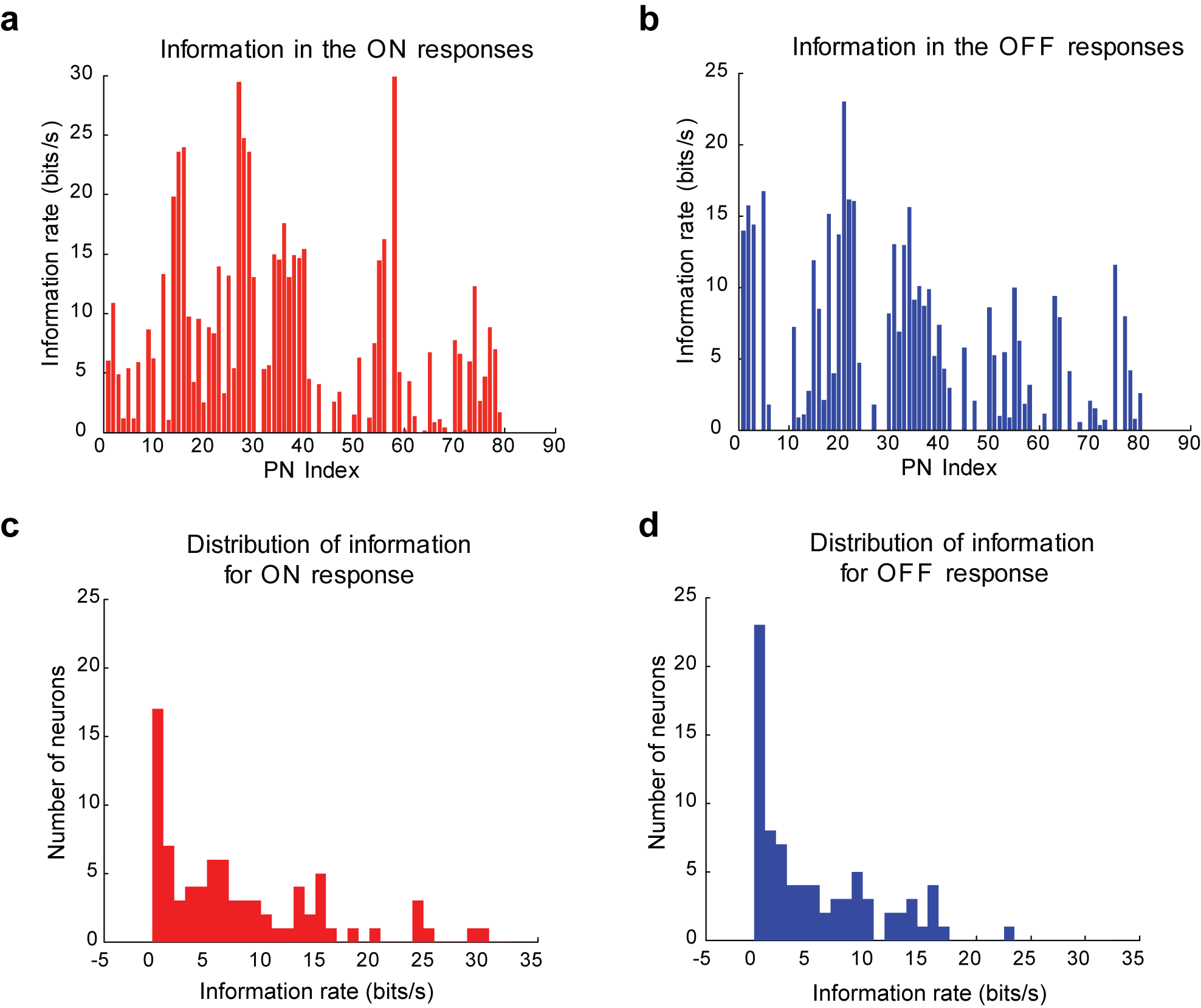


**Supplementary Figure 3: Information theoretic analysis of ON and OFF responses. (a)** The estimated information rate for ON response is plotted for 80 neurons (hex-2oct odor pair). Responses of each neuron to five unique stimuli were used to estimate the total entropy (see Methods). The variations observed in the twenty five repeated trials of the same stimulus were used to estimate the noise entropy. **(b)** Estimated information rate for the OFF response is plotted for the same 80 neurons. **(c, d)** Histograms of ON and OFF information rate distributions are plotted, respectively.


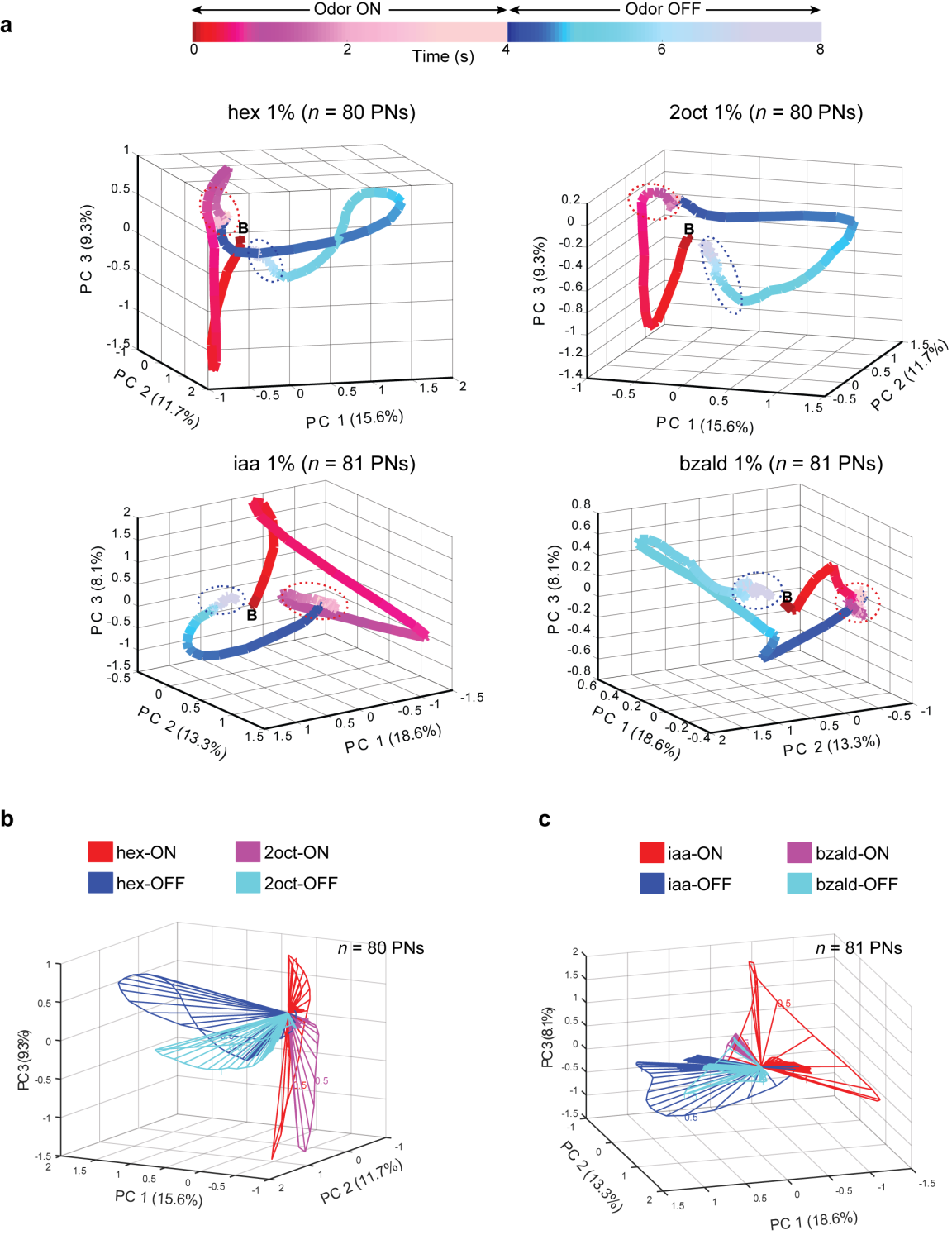


**Supplementary Figure 4: Temporal evolution of odor-evoked ON and OFF responses. (a)** Ensemble projection neuron responses are visualized after the dimensionality reduction using principal component analysis. Each axis corresponds to one of the top three principal components that best captures the variance in the dataset. Percentages of variance captured are shown along each axis. The color bar shown on top reveals how time since odor onset is represented in these trajectory plots. “B” indicates the baseline or pre-stimulus activity. In all panels, steady-state ON responses and OFF responses are identified using red and blue dotted circles, respectively. PN response trajectories during the ON and OFF durations are plotted for the following four odorants: hex 1%, 2oct 1%, iaa 1% and bzald 1%. **(b)** Population response trajectories of PNs (*n* = 80) are plotted after the dimensionality reduction using PCA. Both ON and OFF responses are shown for two odorants: hexanol and 2-octanol. Percentages of variance captured are shown along each axis. **(c)** ON and OFF response trajectories of isoamyl acetate and benzaldehyde are shown.


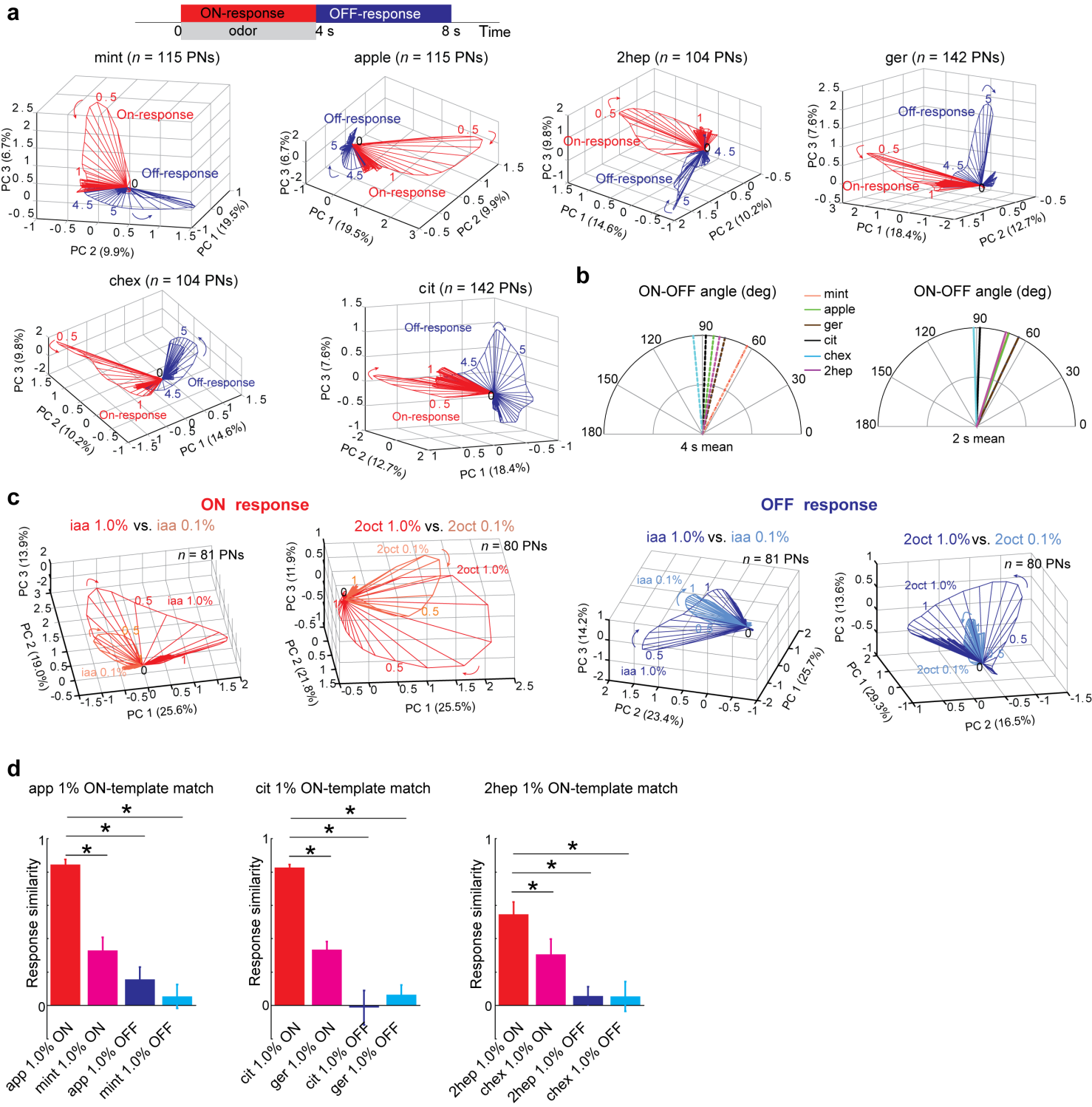


**Supplementary Figure 5: Odor-evoked ON vs. OFF neural responses are nearly orthogonal. (a)** Odor-evoked ensemble PN response trajectory plots are shown for six additional odors (similar plots as in **Fig. 1b**, data re-analyzed from a published study^1^). **(b)** Angular distance between the mean ensemble ON and OFF responses of olfactory PNs is shown (similar to the plots in **Fig. 1f**). **(c)** Ensemble PN ON and OFF response trajectories are shown for two different concentrations of 2oct and iaa (1.0% and 0.1% v/v). Similar odor trajectory plots as shown in **Fig. 2b. (d)** Left, the response similarity (see Methods) between the apple 1% ON template with apple 1% ON, mint 1% ON, apple 1% OFF, and mint 1% OFF are shown (mean ± s.d.). Asterisks indicate significant changes in similarity (**P*<0.05, paired t-test, *n* = 10 trials). Similar plots are shown on the right for cit 1% vs. ger 1% and 2hep 1% vs. chex 1%. Data was re-analyzed from a previous study^1^. For comparisons between ON responses of the same odorant (i.e. first bar on each panel), a leave on trial out validation approach was used.


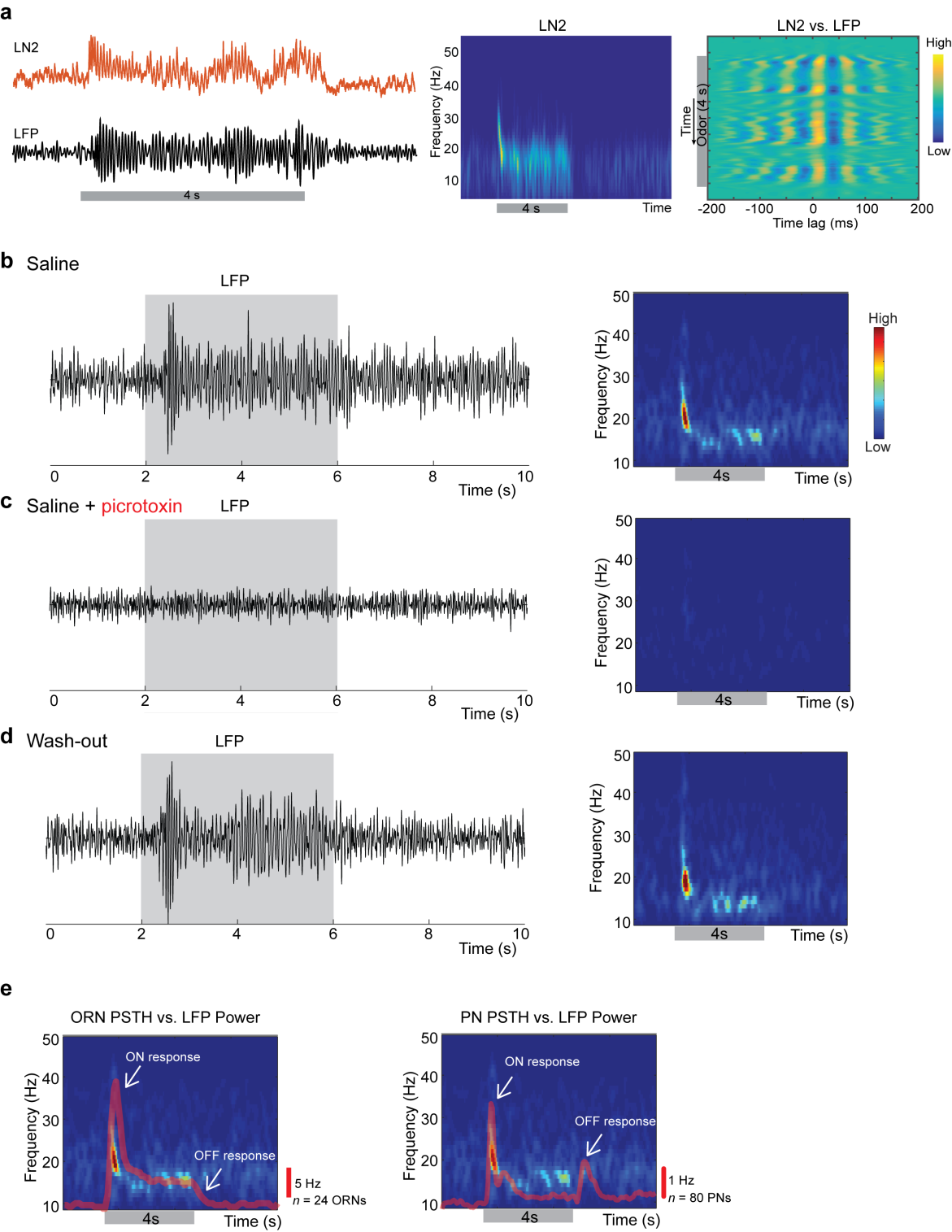


**Supplementary Figure 6: Engaging and disengaging recurrent inhibitory network. (a)** Left, Intracellular response of a local neuron (LN2) and simultaneously recorded extracellular local filed potential (LFP) are shown for a 4 s long odor stimulation. Middle, power at different frequencies are shown for a LN before, during, and after an odor puff. Right, Cross-correlations calculated between the local neuron membrane fluctuations (LN2) and the local field potentials are shown. **(b)** Left, LFP oscillations are shown for a 4 s hexanol puff (control case; before picrotoxin bath application). Right, trial averaged frequency spectrogram is shown for the same odor stimulation (*n* = 3 trials; see Methods). **(c)** Same results are shown when 100 µM picrotoxin was applied in saline bath to block the GABAergic local neurons’ input to the PNs. **(d)** The LFP oscillations and the oscillatory power during odor presentation window recover after saline wash. **(e)** Left, mean ORN firing rate plot is superimposed on the frequency spectrogram shown in panel a (*n* = 24 ORNs). Right, same plot as in left panel but with mean PN firing rate superimposed (*n* = 80 PNs).


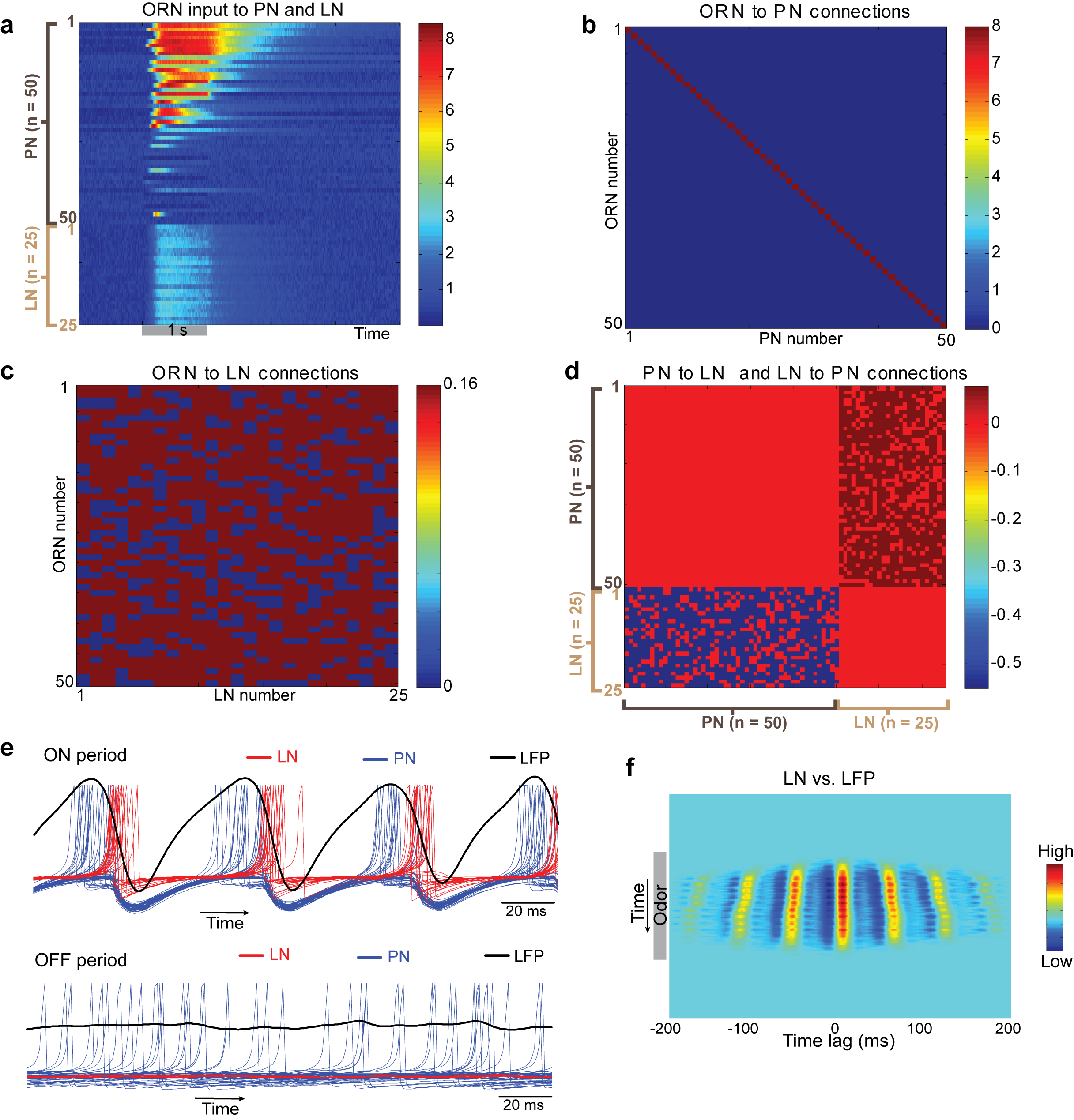


**Supplementary Figure 7: Characterization of the antennal lobe computational model responses and connectivity. (a)** Modeled ORN activity that were input to the PNs and LNs is shown. **(b)** Connectivity matrix between the modeled ORNs and PNs is shown. **(c)** ORN to LN connectivity matrix is shown. **(d)** Connectivity matrix between the PNs and the LNs is schematically shown. **(e)** Top, stimulus-evoked PN (*n* = 50) and LN (*n* = 25) spikes are shown along with the LFP (black) during a 200 ms time window during stimulus exposure. Note that the PN and LN spikes are phase-locked with the LFP. Bottom, similar plots as in top panel but now showing PN, LN spikes and LFP responses during OFF period (after odor termination). Note that both LN spikes and LFP oscillations are absent during this time window although PN spiking activity persists. **(f)** Cross-correlations between LN activity (averaged across all LNs in the antennal lobe model) and the LFP signal are shown. As can be noted, the LN activity transiently phase locked to the LFP during odor presentation window. The red and blue banding patterns indicate the peaks and troughs of the cross-correlation, respectively.

**
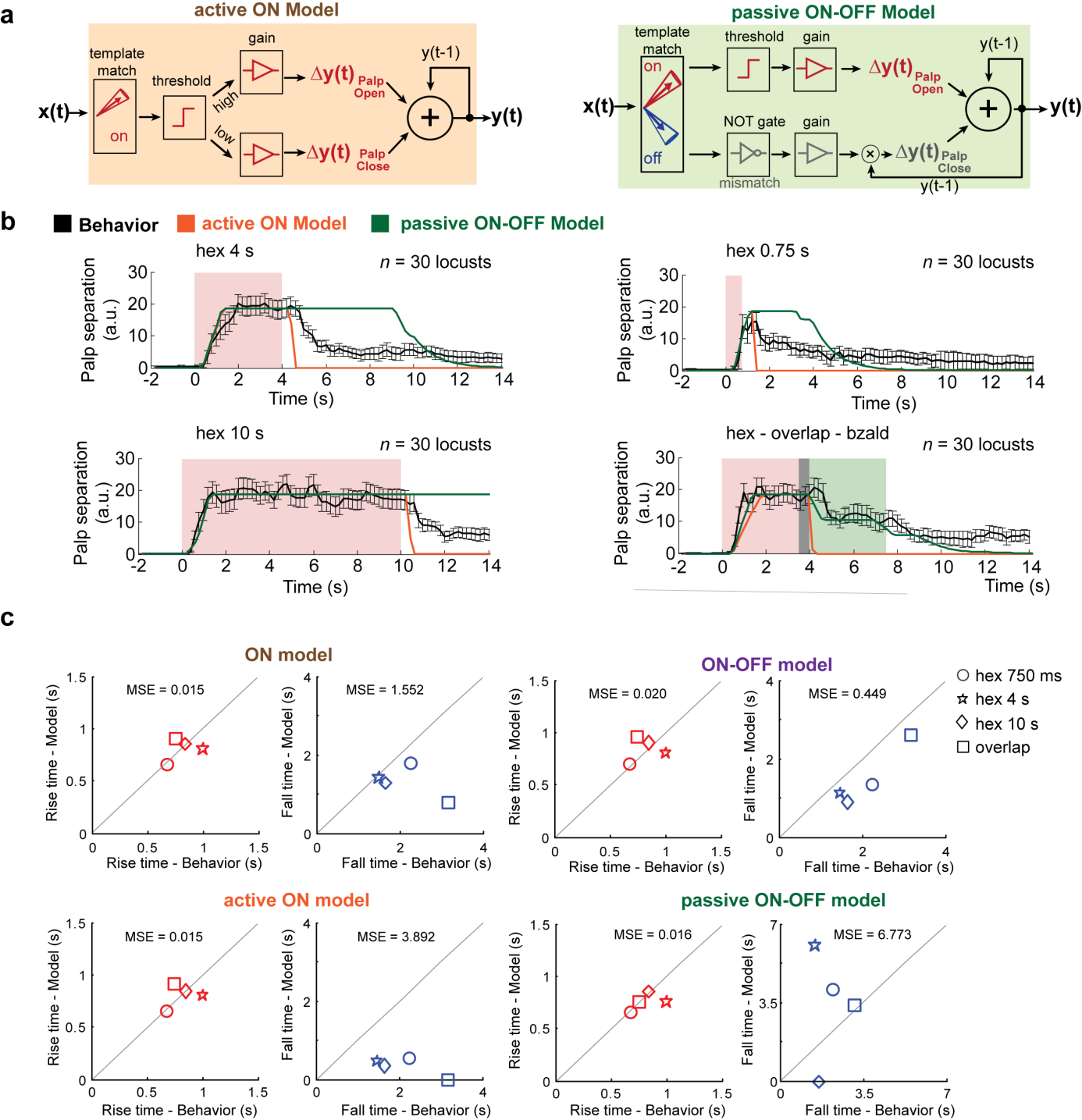
**

**Supplementary Figure 8: Behavioral relevance of ON vs. OFF responses. (a)** Two variants of the models shown in **Fig. 8b** to translate ensemble neural activity to palp opening and closing responses. **Active** **ON model** (*left panel*)**:** A strong pattern-match with the hexanol ON response template initiates and sustains palp-opening response, whereas a weak pattern-match with the ON response template causes a palp-closing response. **Passive** **ON-OFF model** (*right panel*)**:** Pattern-match with ON response template opens and sustains the palps and a lack of pattern-match with both the ON and the OFF responses are necessary to close the palps. **(b)** Comparison of the observed behavioral responses in four different stimulus conditions with the responses predicted by the active ON model and passive ON-OFF model are shown (see Methods). **(c)** Comparisons between the rise-time and the fall-time constants obtained using the responses predicted by each model (y axis) and the actual POR responses obtained from behavioral experiments (x axis) are shown.


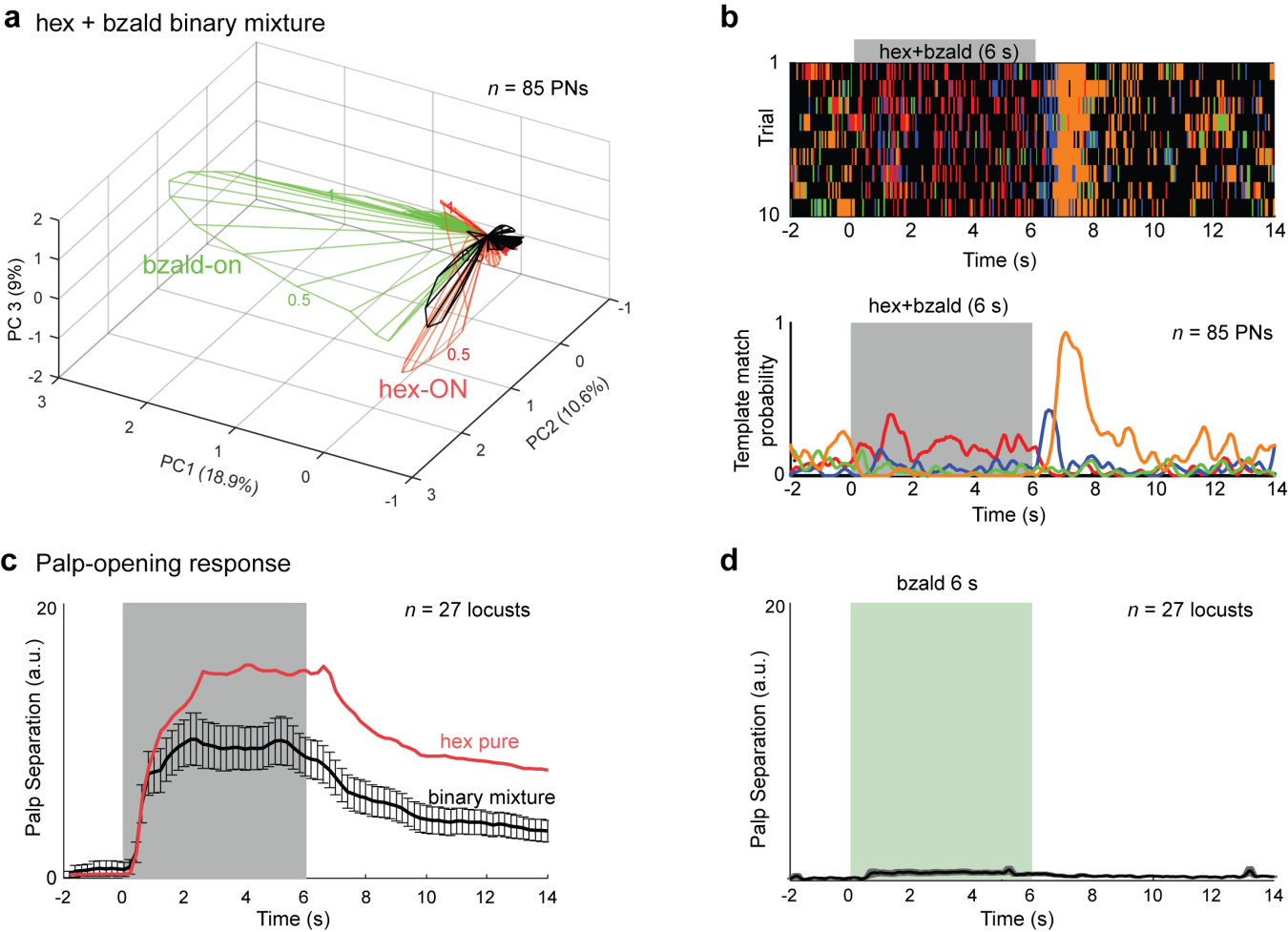


**Supplementary Figure 9:** **Neural and behavioral responses to a binary mixture and its components.** **(a)** Neural trajectory for the mixture of hex and bzald where both components were introduced simultaneously (note only ON responses are shown for clarity). The synchronous binary mixture of hex-bzald generated population neural responses that were aligned more with the hex-ON response alone. This new data was collected from a different PN population as compared to **Fig. 5**. **(b)** Results from a bin-by-bin, trial-by-trial classification analysis are shown for the synchronous mixture of hex and bzald. The ON and OFF responses observed during solitary hex and bzald introductions were used as templates to be pattern-matched. Bottom, the probabilities of pattern-match with different response templates are shown as a function of time. **(c)** Median palp-opening responses are shown for a 6 s hexanol presentation (CS, in red) and the synchronous binary mixture (hex+ bzald, in black). Error bars represent s.e.m. Total of 27 locusts were tested for this study. **(d)** No significant palp opening response was observed when a 6 s bzald odorant was puffed solitarily.


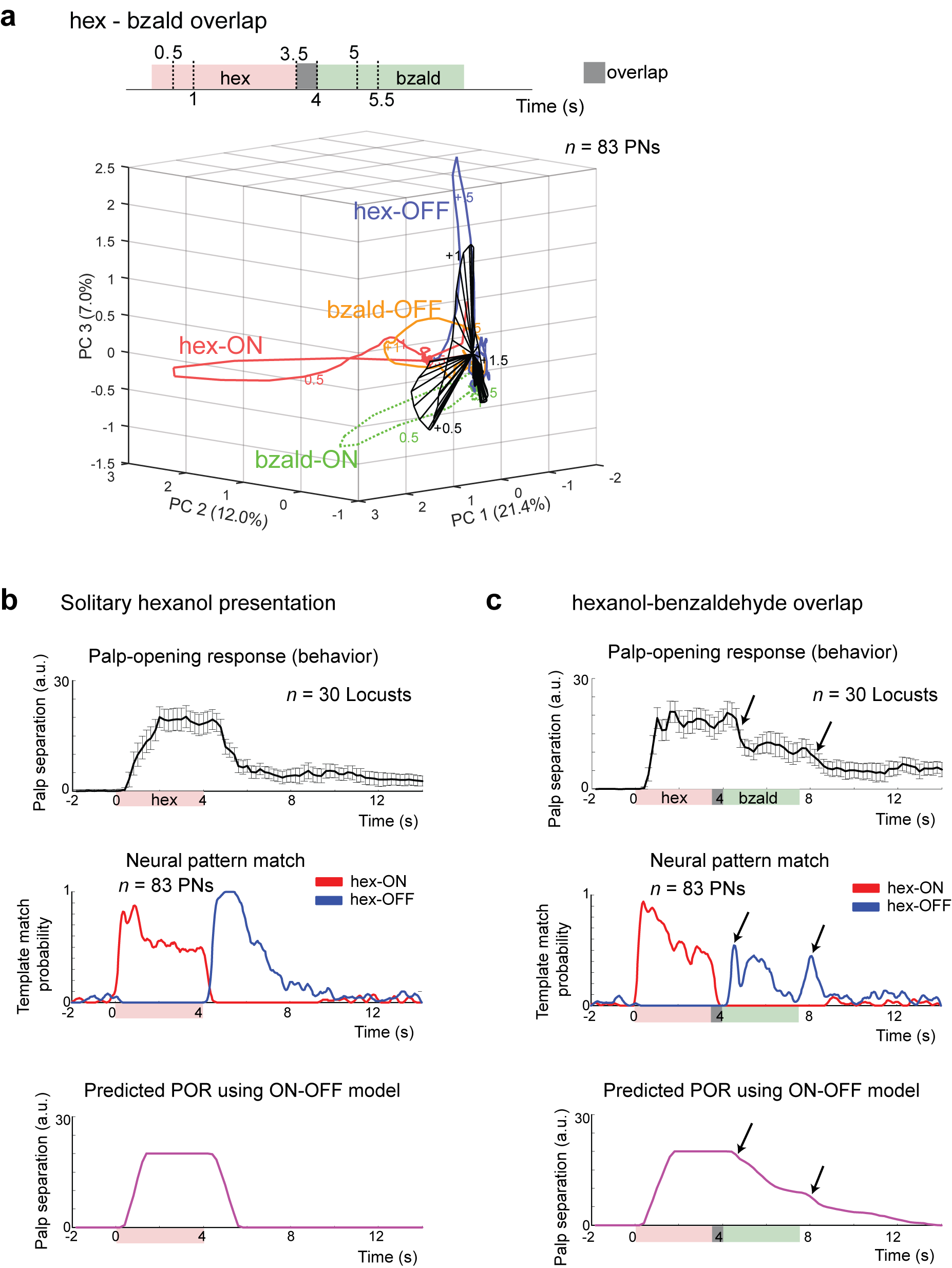


**Supplementary Figure 10: ON-OFF model a better predictor of POR. (a)** Neural response trajectory evoked by the overlapping sequence of hex-bzald is shown. The black trajectory shows the 4 s period following bzald application. The response trajectories elicited by solitary presentations of hex and bzald are also shown to facilitate comparisons. Color-coded numbers on the plot indicate time since introduction of a particular odorant. *n* represents the number of neurons used for this analysis. **(b)** Top, the median palp opening response is plotted (± s.e.m; *n* = 30 Locusts) in the case of 4 s duration hexanol presentation. Middle, the ensemble neural response match with the hex-ON and hex-OFF template are shown during the odor onset and offset periods. Bottom, the predicted POR using the ON-OFF model (see Methods) is shown for 4 s hexanol solitary presentations. **(c)** Same format as in **panel b** but results are shown for the hex-0.5s overlap-bzald odor sequence.

**Reference:**

1 Saha, D. *et al.* A spatiotemporal coding mechanism for background-invariant odor recognition. *Nat Neurosci* **16**, 1830-1839 (2013).
